# Assessing the Effectiveness of Curative Benznidazole Treatment in Preventing Chronic Cardiac Pathology in Experimental Models of Chagas Disease

**DOI:** 10.1128/AAC.00832-18

**Published:** 2018-09-24

**Authors:** Amanda Fortes Francisco, Shiromani Jayawardhana, Martin C. Taylor, Michael D. Lewis, John M. Kelly

**Affiliations:** a Department of Pathogen Molecular Biology, London School of Hygiene and Tropical Medicine, London, United Kingdom

**Keywords:** Chagas disease, benznidazole, cardiac pathology, experimental model

## Abstract

Chagasic heart disease develops in 30% of those infected with the protozoan parasite Trypanosoma cruzi, but can take decades to become symptomatic. Because of this, it has been difficult to assess the extent to which antiparasitic therapy can prevent the development of pathology. We sought to address this question using experimental murine models, exploiting highly sensitive bioluminescent imaging to monitor curative efficacy. Mice were inoculated with bioluminescent parasites and then cured in either the acute or chronic stage of infection with benznidazole. At the experimental endpoint (5 to 6 months postinfection), heart tissue was removed and assessed for inflammation and fibrosis, two widely used markers of cardiac pathology. Infection of BALB/c and C3H/HeN mice with distinct T. cruzi lineages resulted in greatly increased myocardial collagen content at a group level, indicative of fibrotic pathology. When mice were cured by benznidazole in the acute stage, the development of pathology was completely blocked. However, if treatment was delayed until the chronic stage, cardiac fibrosis was observed in the BALB/c model, although the protective effect was maintained in the case of C3H/HeN mice. These experiments therefore demonstrate that curative benznidazole treatment early in murine T. cruzi infections can prevent the development of cardiac fibrosis. They also show that treatment during the chronic stage can block pathology but the effectiveness varies between infection models. If these findings are extendable to humans, it implies that widespread chemotherapeutic intervention targeted at early-stage infections could play a crucial role in reducing Chagas disease morbidity at a population level.

## Abstract (precis)

Chagasic heart disease develops in 30% of those infected with the protozoan parasite Trypanosoma cruzi, but can take decades to become symptomatic. Because of this, it has been difficult to assess the extent to which antiparasitic therapy can prevent the development of pathology.

## INTRODUCTION

Chagas disease results from infection with the protozoan parasite Trypanosoma cruzi, and it is an important public health problem throughout Latin America (1, 2). A total of 5 to 8 million people are infected, mainly in poor rural areas. Hematophagous triatomine bugs are the principal route of transmission, although contaminated food and drink, blood transfusion, and the congenital route are also important. As a result of migration, many cases of Chagas disease are now being diagnosed outside regions where it is endemic, particularly in the United States and Europe (3, 4).

T. cruzi is an obligate intracellular parasite and can infect most nucleated cells. During the acute stage of the disease (2 to 8 weeks postinfection in humans), parasites become widely disseminated in tissues and organs and can be detected in the bloodstream. In the majority of cases, the acute stage results in a mild febrile illness. Occasionally, particularly in children, the outcomes can be more serious, and sometimes fatal, due to myocarditis or meningoencephalitis. In most individuals, the infection is controlled by the adaptive immune response driven by CD8^+^ IFN-γ^+^ T cells (5, 6), and the disease then progresses to an asymptomatic chronic stage, which is characterized by an extremely low parasite burden. However, over time (sometimes decades), 30% of those infected develop pathology, which can manifest as cardiomyopathy and sudden heart failure (7–9). In addition, about 10% develop digestive tract megasyndromes, symptoms that sometimes overlap with the cardiac complications.

The nitroheterocyclic drugs benznidazole and nifurtimox have been used to treat Chagas disease for more than 50 years (10, 11). However, they have a range of side effects and require extended dosing (60 to 90 days) (12–14). As a result, compliance is a problem and treatment failure is common. There is also potential for cross-resistance, since both drugs require bioactivation within the parasite by the mitochondrial nitroreductase TcNTR-1 (15, 16). Despite these issues, clinical trials have demonstrated that parasitological cure is achievable in both the acute and chronic stages (12, 13, 17, 18). However, the capacity of curative drugs to prevent or alleviate cardiac pathology is unresolved. Addressing this question is one of the major challenges in the Chagas disease field.

Cardiomyopathy in chronic Chagas disease is associated with inflammation, fibrosis, thromboembolism, ventricular arrhythmia, and progressive cardiac failure (19, 20). The mechanisms that drive pathology have been the subject of vigorous debate focused on the central question of whether parasite persistence is required. There has been a long-standing hypothesis that autoimmunity has a crucial role. This is based on numerous reports of cross-reactivity between epitopes in host and parasite proteins, the rapid polyclonal expansion of B and T cells during the acute stage, including some that are autoreactive (21, 22), and the inability to consistently detect parasites in chagasic hearts. Resolving the importance of parasite persistence in humans is complex because of the long period over which chronic pathology develops and the highly diverse nature of the disease. Furthermore, factors such as host and parasite genetics and immune status are also thought to have a crucial influence (23). Identifying the underlying mechanisms responsible for pathology has major implications for the management and treatment of chronic T. cruzi infections.

In the absence of robust experimental systems for investigating pathogenesis in humans, predictive animal models have an important role in Chagas disease research. Several mouse models are available that display symptoms analogous to those in humans, particularly in terms of cardiac pathology. However, practical difficulties in detecting the extremely low numbers of parasites in the chronic stage have complicated the establishment of a definitive link between curative treatment and the prevention of clinical disease. Recently, highly sensitive *in vivo* bioluminescence imaging applicable to chronic murine infections has provided an improved framework for addressing this question (24, 25). Studies have revealed that parasites are readily detectable in most organs and tissues during the acute stage (24, 26). As the infection proceeds, the adaptive immune response then reduces the parasite burden by up to 3 orders of magnitude, although the infection is not eliminated. The gastrointestinal (GI) tract is the major parasite reservoir during the chronic stage, with infection of other organs being sporadic and influenced by host and parasite genetics. These observations are consistent with a model in which the gut provides an immunotolerant niche that permits long-term maintenance of the infection, with intermittent trafficking of parasites or parasite-infected cells to other sites (27). Crucially, cardiac damage in these mice was detectable in the absence of locally persistent infection (24, 26). This implies that episodic infections of the heart may give rise to localized immune responses that clear the parasite, but which result in cumulative tissue damage.

There is now a general view that the presence of the parasite is required for the development of chagasic heart disease (28). If correct, this suggests that curative drug treatment should have a beneficial outcome in terms of pathology. Several recent reports focused on acute stage murine infections seem to support this (29–33). With chronic-stage infections the data are less clear-cut (34), in part because it is difficult to confirm parasitological cure, even with PCR-based methodologies. In humans, the BENEFIT trial reported no obvious improvements in cardiomyopathy-related clinical outcomes 5 years after chronic stage infections were treated with benznidazole (17). However, since patients for this study were selected on the basis of preexisting cardiomyopathy, it has not been possible to extrapolate the findings to the majority of chronically infected individuals, who are asymptomatic.

New information on the extent to which parasitological cure impacts on the progression of heart pathology is crucial for informing the international effort aimed at developing new drugs against Chagas disease. Here, we have exploited predictive murine models and highly sensitive bioluminescence imaging to monitor curative treatment at various stages of the disease cycle and to assess the outcomes in terms of cardiac pathology.

## RESULTS

**FIG 1:**
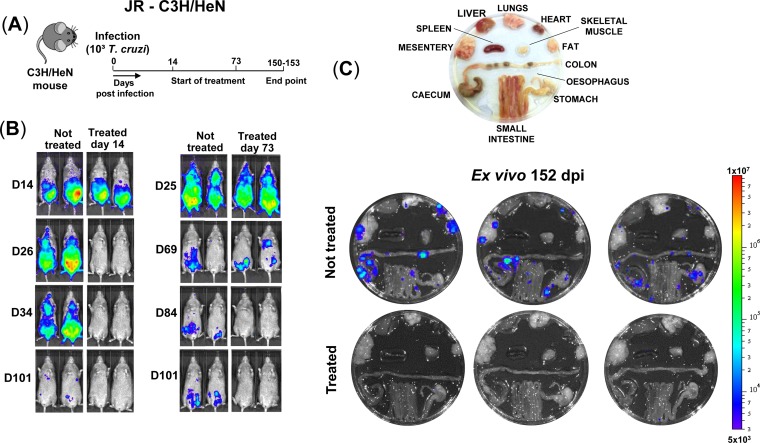
Benznidazole treatment in both the acute and chronic stage cures C3H/HeN mice infected with T. cruzi JR strain. (A) Outline of infection and treatment schedule. Mice were treated with 100 mg/kg benznidazole once daily by the oral route for 20 days (Materials and Methods). (B) Representative ventral *in vivo* images of treated (starting day 14 or 73) and nontreated mice at sequential time points postinfection. All images use the same log_10_-scale heat-map with minimum and maximum radiance values as indicated. (C) *Ex vivo* bioluminescence images of organs and tissues taken at 152 days postinfection (dpi). Organs arranged as shown (upper image).

**FIG 2:**
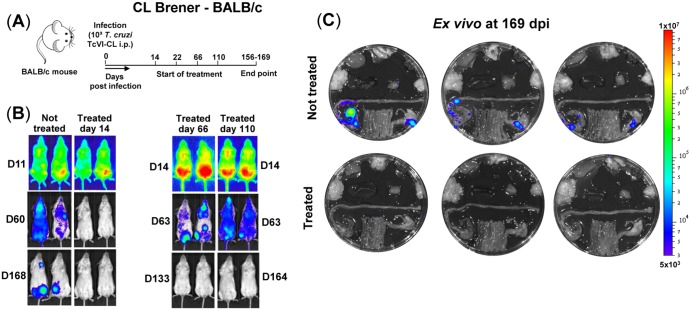
Benznidazole treatment in both the acute and chronic stage cures BALB/c mice infected with T. cruzi strain CL Brener. (A) Outline of infection and treatment schedule. Mice were treated with 100 mg/kg benznidazole once daily by the oral route for 20 days (see Materials and Methods). (B) Representative ventral *in vivo* images of treated (starting day 14, 66, or 110) and nontreated mice at sequential time points postinfection. (C) *Ex vivo* bioluminescence images of organs and tissues taken at 169 days postinfection (dpi). Organs arranged as shown in Fig. 1C.

Our aim was to investigate if treatment with curative doses of benznidazole prevented or alleviated the development of chagasic cardiac damage in mice by assessing myocarditis and myocardial fibrosis, widely used quantifiable markers of pathology. To reflect the genetic diversity of T. cruzi, we selected strains JR (discrete typing unit [DTU] I) and CL Brener (DTU VI), both of which were modified to express a red-shifted luciferase (24, 26). Following inoculation into the peritoneal cavity, the limit of detection with this highly sensitive system is close to 100 parasites, and there is a strong correlation between total body bioluminescence and parasite burden (24). Two infection combinations were investigated, strain JR in C3H/HeN mice (JR-C3H) and strain CL Brener in BALB/c mice (CL-BALB/c). Previously, we have shown that chronic infections in these models give rise to significant pathological fibrosis, which is particularly severe in the case of the JR-C3H combination, although endpoint cardiac inflammation tends to be mild or negligible (24, 26). To initiate infections, mice were inoculated intraperitoneally (i.p.) with 1 × 10^3^ T. cruzi bloodstream trypomastigotes. At various time points postinfection, they were treated with benznidazole at 100 mg/kg for 20 days (35), a regimen that cures both acute and chronic infections (Fig. 1 and 2).

Curative benznidazole treatment of infected C3H mice was initiated during the acute stage (day 14) or after transition to the chronic phase (day 73) (Fig. 1A). Mice were then monitored by *in vivo* imaging until the experimental endpoint (days 150 to 153). In nontreated mice, the infection reached a peak approximately 21 days postinfection. This was followed by progressive reduction in the parasite burden until the infection entered the chronic stage (days 50 to 60), when levels were up to three orders of magnitude lower (Fig. 1B). The chronic stage is characterized by the presence of highly dynamic bioluminescent foci that are detectable by *in vivo* imaging (26). When the internal organs were examined by *ex vivo* imaging, bioluminescent foci were also detectable in the colon and/or stomach in all cases and sporadically in other organs, including the heart (Fig. 1C). In the JR-C3H model, infection foci were observed more frequently in non-GI tract sites than was the case in other parasite/mouse strain combinations (26), although there was no specific tropism for the heart.

**FIG 3:**
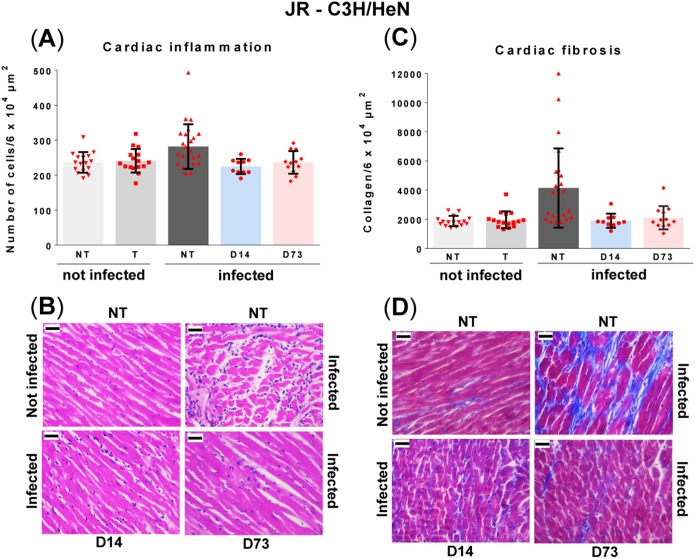
Curative benznidazole treatment of C3H/HeN mice infected with T. cruzi strain JR prevents the development of cardiac fibrosis. Infected mice (see Materials and Methods) were either nontreated (NT; *n* = 24), or treated (T) with benznidazole for 20 days, as outlined in the legend to Fig. 1, commencing day 14 postinfection (*n* = 10), or day 73 (*n* = 12). Groups of noninfected mice, either nontreated (NT; *n* = 16), or treated (T) (starting on day 14, *n* = 8, or on day 73, *n* = 8) were run in parallel. Cardiac tissue was harvested (day 150 to 153) and assessed for inflammation and fibrosis (see Materials and Methods). (A) Quantitative histopathological analysis of inflammation. The number of nuclei per 6 × 10^4^ μm^2^ was quantified as a cellular myocarditis index. Data are shown as mean ± standard deviation. Inflammation in nontreated infected mice was slightly but significantly greater than that in noninfected controls or in drug cured mice. (B) Representative myocardial sections stained with H&E and used to assess inflammation. (C) Quantification of collagen content (blue area in Masson's trichrome-stained sections) as a marker of cardiac fibrosis (see the text for more details and Materials and Methods for description of statistical procedures). (D) Masson's trichrome-stained photomicrographs demonstrating fibrosis in mice heart sections. In panels B and D, the magnification is 400×. Bar, 30 μm.

To evaluate the capacity of curative treatment (Fig. 1B and C) to prevent pathology, quantitative analysis of inflammation and fibrosis was performed on cardiac muscle obtained 150 to 153 days postinfection. There was a slight increase in myocardium cellularity in nontreated infected C3H mice compared to age-matched noninfected, nontreated controls, although this was just below the level of significance (*P* = 0.13) (Fig. 3A and B). No parasites were detected in any cardiac images from nontreated animals (∼360 examined), although bioluminescence signals suggested low-level infections in some mice (Fig. 1C). Exposure to benznidazole in the absence of infection did not cause myocarditis (Fig. 3A). In mice cured in either the acute or chronic stage of infection, cardiac inflammation was similar to that in the noninfected controls and displayed no significant correlation with fibrosis at the level of individual mice (below).

We used collagen content as an indicator of pathological cardiac fibrosis (see Materials and Methods). Baseline levels in noninfected control mice displayed considerable uniformity, with no significant differences between benznidazole-treated and nontreated animals (Fig. 3C and D). In contrast, in nontreated infected mice, there was remarkable variation, with differences of more than 5-fold in the extent of fibrosis within the cohort (24 mice, 15 cardiac images examined per individual). At a group average level, cardiac collagen was more than 2-fold higher in the nontreated infected mice than in the noninfected mice (*P* = 0.0006). However, within this group comparison, approximately half of the JR-infected mice exhibited no evidence of fibrosis, whereas the other half displayed major increases in collagen content (Fig. 3C). When C3H mice that had been cured of T. cruzi infection with benznidazole were evaluated at the experimental endpoint (day 150 to 153), the myocardial collagen content was found to be considerably lower than in the noncured group (treatment initiation on day 14, *P* = 0.008; on day 73, *P* = 0.03) and not significantly different from that in noninfected mice (Fig. 3C and D). These data therefore suggest that in the JR-C3H model, curative drug treatment in either the acute or chronic stage of infection can prevent the development of cardiac fibrosis.

**FIG 4:**
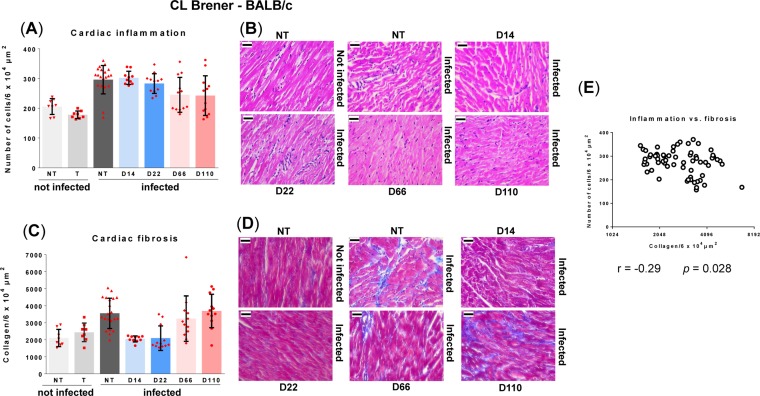
Curative benznidazole therapy prevents the development of cardiac fibrosis in BALB/c mice infected with T. cruzi CL Brener when treatment is initiated in the acute stage but not when initiated in the chronic stage. Infected mice were either nontreated (NT; *n* = 19), or treated (T) with benznidazole for 20 days, as outlined in the legend to Fig. 2, commencing day 14 postinfection (*n* = 10), day 22 (*n* = 12), day 66 (*n* = 12), or day 110 (*n* = 12). Groups of noninfected mice, either nontreated (NT; *n* = 7), or treated (T) (starting on day 66, *n* = 4, or on day 110, *n* = 4) were run in parallel. Cardiac tissue was harvested and assessed for inflammation and fibrosis (see Materials and Methods). (A) Quantitative histopathological analysis of inflammation. The number of nuclei per 6 × 10^4^ μm^2^ was quantified as a myocarditis index. Data are shown as mean ± standard deviation. (B) Representative myocardial sections stained with H&E demonstrating inflammation. (C) Quantification of collagen content (blue area in Masson's trichrome-stained sections) as a marker of cardiac fibrosis. (D) Masson's trichrome-stained photomicrographs demonstrating fibrosis in mice heart sections. In panels B and D, the magnification is 400×. Bar, 30 μm. (E) Pearson correlation analysis of myocarditis against cardiac fibrosis scores. Data are from samples obtained at 169 days postinfection from the groups shown in panels A and C.

When the CL-BALB/c parasite-mouse combination was used to further examine the effect of curative drug-treatment (Fig. 2B and C) on chronic cardiac pathology, we included additional treatment time points in the acute and chronic stages (Fig. 2A). In this model, the acute-stage parasite burden peaks at day 14 postinfection, but the subsequent profile of parasite infection is otherwise generally similar to that in the JR-C3H combination. In line with previous results (26), we observed that during BALB/c chronic stage infections, CL Brener parasites were more tightly restricted to the GI tract than was the case with JR infections of C3H mice (Fig. 1C and 2C). Histopathological analysis of cardiac tissue from nontreated CL Brener-infected BALB/c mice (days 156 to 169 postinfection) revealed significant diffuse cellular infiltration compared to that in noninfected controls (Fig. 4A and B) (*P* = 0.005). Inflammatory infiltrates were comprised predominantly of mononuclear cells with lymphocytic morphology. Similarly, an increase in inflammatory infiltration was observed in mice that had been cured with benznidazole during the acute stage (treatment starting day 14 or 22; *P* = 0.0004 and *P* = 0.005, respectively). Mice that were cured during the chronic stage showed some signs of myocarditis, but infiltrates were less intense and below the level of significance (treatment starting days 66 and 110; *P* = 0.26 and *P* = 0.44, respectively).

In nontreated BALB/c mice infected with CL Brener, we found a significant increase in fibrosis compared to that of age-matched noninfected controls (Fig. 4C and D) (*P* = 0.007) when cardiac sections were examined at the experimental endpoint. However, as with the JR-C3H combination, there was considerable variation in the extent of fibrosis following infection and only a minor or negligible correlation with inflammation (Fig. 4E). In both models, collagen deposits were diffusely distributed within the muscle fibers and interstitial spaces, although in the JR-C3H combination, fibrosis was more often present as dense focal deposits organized between muscle fibers. This intense fibrosis in the interstitial spaces produced larger gaps between muscle fibers. No differences in inflammation or fibrosis between atrial and ventricular regions of the heart were apparent. When cardiac sections from mice cured in the acute stage (treatment starting day 14 or 22) were examined on days 156 to 169, there was no evidence of fibrosis, as the level of collagen was considerably less than that in the nontreated cohort (Fig. 4C) (*P* = 0.0017 and *P* = 0.0002, respectively). Levels were the same as those in the noninfected controls, a pattern similar to that in the JR-C3H combination (Fig. 3C). However, in samples taken from mice treated in the chronic stage, cumulative fibrosis was apparent. The collagen content was significantly greater in mice where treatment was initiated on day 110 (*P* = 0.009), similar to that in noncured mice (Fig. 4C). When treatment began on day 66, several of the mice exhibited fibrosis at the experimental endpoint, although this increased collagen content did not reach the level of significance at a group level (*P* = 0.30). Therefore, in the CL-BALB/c experimental model, the timing of curative treatment during an infection is crucial for preventing cardiac pathology.

## DISCUSSION

Chagas disease drug discovery programs are predicated on the assumption that antiparasitic therapy can prevent or reduce the development of chronic pathology. The target group for these drugs is the 5 to 8 million individuals in the asymptomatic chronic phase of the disease, since the vast majority of acute-stage infections pass undetected and patients with advanced symptoms are unlikely to benefit (17). Increasing evidence suggests that parasite persistence is a prerequisite for cardiac pathology (27, 28, 36, 37), providing a theoretical rationale for curative therapy. Despite this, it has been problematic to confirm beneficial outcomes in humans (17). Predictive experimental models are therefore of particular importance. However, even in mice, studies into the effectiveness of antiparasitic treatment have been confounded by a general inability to monitor infections during the chronic stage and by difficulties in demonstrating sterile cure.

In the current work, we exploited highly sensitive bioluminescent imaging to confirm antiparasitic drug efficacy (Fig. 1C and 2C). First, we assessed the capacity of benznidazole to prevent pathology in the JR-C3H parasite-host combination. Previous studies revealed considerable variation in the severity of cardiac fibrosis during chronic infections in this model (26). Large cohort sizes were therefore used to provide statistical power. As expected, we found extensive differences in the level of collagen deposition in the heart muscle of infected nontreated mice (Fig. 3C and D). This heterogeneity is typical of cardiac pathology in humans during chronic Chagas disease. Intriguingly, curative treatment during both the acute and chronic stage of infection completely blocked the development of fibrosis. We then used the CL-BALB/c combination, and we included additional experimental time points to better assess the protective effect. In this model, chronic infection also led to significantly increased fibrosis in the untreated mice (Fig. 4C and D), and antiparasitic treatment during the acute stage (starting days 14 or 22) prevented pathological collagen deposition in cardiac muscle. However, if treatment was delayed until the chronic stage (starting day 110), high levels of fibrosis were observed, similar in severity to those in nontreated mice.

In murine models of chronic Chagas disease, the GI tract is the major site of parasite persistence (25, 26), with sporadic infection of the heart and other organs (e.g., Fig. 1C and 2C). The heart does not appear to be preferentially infected, and fibrosis can develop in the absence of cardiac-localized parasite persistence. We have proposed that pathology arises from collateral damage resulting from immune responses triggered by long-term, intermittent reinvasion of the heart by parasites derived from more permissive niches, which may include the GI tract (27). In both mice and humans, this dynamic process could contribute to the heterogeneous and cumulative nature of pathology, with the low regenerative capacity of cardiac muscle (38) accounting for the organ-specific damage.

The major finding of our current study is that curative treatment can prevent cardiac fibrosis. In the case of the CL-BALB/c model, this requires treatment earlier in the infection, whereas with the JR-C3H combination, treatment can be postponed until at least day 73 without compromising the protective effect. This disparity could reflect temporal variations in the infection profile between the two models, the type of localized immune response, parasite diversity, or a combination of all three. It would be premature at this stage to extrapolate these differences and to define them as representative of specific parasite lineages. In the case of myocarditis, there was not a significant association with infection in the JR-C3H model (Fig. 3). Although this was more pronounced in the CL-BALB/c combination, there was no positive correlation between endpoint inflammation and fibrosis at the level of individual mice (Fig. 4E). This is perhaps unsurprising given the episodic character of active heart parasitism and snapshot nature of the endpoint analysis. We have observed this lack of association previously and found that it is the inability to restrict infection to the gut during chronic infections that best correlates with heart disease severity (26).

The findings reported here demonstrate that antiparasitic treatment can prevent cardiac damage, and that T. cruzi persistence, even when not continuous in the heart, is a major determinant of fibrosis. Of particular significance, is our finding in the CL-BALB/c model that delays in administering curative treatment can have detrimental effects on disease outcome, suggesting that pathology is cumulative in the presence of infection. Our hypothesis is that pathology results from collateral damage caused by the localized immune responses that eradicate the intermittent cardiac infections during the chronic stage. The apparent stochastic nature of these infections may contribute to the diversity of pathological outcomes, although other factors are almost certainly involved. In this context, we tried to capture some of the potential variation in disease outcome attributable to host:parasite strain differences by using the T. cruzi CL Brener (DTU VI) and JR (DTU I) isolates, and the BALB/c and C3H/HeN mouse strains. More detailed studies in this area are clearly warranted to further dissect the effect of host and parasite diversity on disease pathology.

If the results reported here are applicable to humans, they strongly support a policy of early treatment of individuals who are seropositive for T. cruzi, but asymptomatic. This could prevent the time-dependent build-up of largely irreversible cardiac damage. At present, up to 99% of asymptomatic people infected with T. cruzi are unaware of their condition (39). Therefore, population-wide diagnostic programs should be considered a public health priority in areas where Chagas disease is endemic, particularly as more effective drugs become available.

## MATERIALS AND METHODS

### Parasites.

Bioluminescent T. cruzi strains (CL Brener and JR) that constitutively express the red-shifted luciferase PpyRE9h (40) were generated and cultured as described previously (24, 26). Metacyclic trypomastigotes were produced by transfer to supplemented Grace's medium (41) and harvested after 4 to 7 days. Tissue culture trypomastigotes were obtained from infected L6 rat myoblasts (26).

### Murine infections and bioluminescence imaging.

Animal work was performed under United Kingdom Home Office project licenses (PPLs 70/6997 and 70/8207) and approved by the LSHTM Animal Welfare and Ethical Review Board. Procedures were conducted in accordance with the United Kingdom Animals (Scientific Procedures) Act 1986 (ASPA). BALB/c and C3H/HeN mice were purchased from Charles River (UK), and CB17 SCID mice were bred in-house. Animals were maintained under specific pathogen-free conditions in individually ventilated cages. They experienced a 12-h light/dark cycle, with access to food and water *ad libitum*. SCID mice were infected with 1 × 10^4^ bloodstream trypomastigotes (BTs) in 0.2 ml phosphate-buffered saline (PBS) via i.p. injection. BALB/c and C3H female mice (aged 7 to 8 weeks) were infected by i.p. injection of 1 × 10^3^ BTs derived from SCID mouse blood. At experimental endpoints, mice were sacrificed by exsanguination under terminal anesthesia.

For *in vivo* bioluminescence imaging, mice were injected with 150 mg/kg d-luciferin i.p., then anesthetized using 2.5% (vol/vol) gaseous isoflurane (25). Five to 10 min after d-luciferin administration, they were placed in an IVIS Lumina II system (Caliper Life Science), and images were acquired using LivingImage v4.3. Exposure times varied between 30 s and 5 min, depending on signal intensity. After imaging, mice were revived and returned to cages. For *ex vivo* imaging, mice were injected with d-luciferin and sacrificed by exsanguination under terminal anesthesia 5 min later. They were then perfused via the heart with 10 ml of 0.3 mg/ml d-luciferin in PBS. Organs and tissues were removed and transferred to a petri dish in a standardized arrangement (Fig. 1C), soaked in 0.3 mg/ml d-luciferin in PBS, and imaged using maximum detection settings (5 min exposure, large binning). The remaining animal parts and carcass were checked for residual bioluminescent foci, also using maximum detection settings.

To estimate parasite burden in live mice, regions of interest (ROI) were drawn using LivingImage v4.3 to quantify bioluminescence as total flux (photons/s), summed from dorsal and ventral images. The detection threshold for *in vivo* imaging was determined using control uninfected mice. To quantify infection intensities in *ex vivo* tissues, individual ROIs were drawn to quantify bioluminescence expressed as radiance (p/s/cm^2^/sr).

### Drug treatment.

Benznidazole was synthesized by Epichem Ltd. (Australia) and prepared for administration at 10 mg/ml in 5% dimethyl sulfoxide (vol/vol)/95% hydroxypropyl methylcellulose (HPMC) suspension vehicle (0.5% (wt/vol) hydroxypropyl methylcellulose, 0.5% (vol/vol) benzyl alcohol, and 0.4% (vol/vol) Tween 80 in Milli-Q-water). Mice were treated at 100 mg/kg for 20 days by oral gavage (35) (Fig. 1 for details).

### Cardiac histomorphometric studies.

Heart samples were fixed in Glyo-Fixx for 24 h, dehydrated in ethanol, cleared in Histo-Clear, and embedded in paraffin. Three-micron heart sections were stained with hematoxylin and eosin (H&E) or Masson's trichrome (MT). Images covering atrioventricular regions were acquired with a camera (Leica DFC295) attached to a Leica DM3000 light-emitting diode (LED) microscope. Images were digitalized for histomorphometric analysis using the Leica Application Suite v4.5 software. The base of the heart and major vessels were excluded because of inherently high collagen content. The number of images required for accurate representative quantification of the whole myocardium was determined by stability analysis of measurements based on a range of fields, from a minimum of 5 to a maximum of 20. An index of inflammatory cells was assessed by quantifying a standardized test area of 6 × 10^4^ μm^2^ per image, acquired with a 40× objective. The number of cells/image was determined from the average of 15 images/animal at ×400 magnification, randomly chosen, with samples stained with H&E. An increase in the number of cells compared with uninfected controls was considered indicative of inflammation. An index of collagen type I, the most abundant form, which accounts for 90% of total collagen in mammals, was assessed by quantifying a standardized test area of 6 × 10^4^ μm^2^ per image, acquired with a 40× objective. The area in blue MT staining was determined from an average of 15 randomly chosen images/animal, at ×400 magnification. Increased collagen content compared with that of uninfected controls was considered indicative of cardiac fibrosis.

### Statistics.

Individual animals were used as the unit of analysis unless otherwise stated. Statistical differences between groups were evaluated using a Kruskal-Wallis test with Dunn's *post hoc* correction in GraphPad Prism v.7.03. Differences of *P* < 0.05 were considered significant.
